# Delay of innate immune responses following influenza B virus infection affects the development of a robust antibody response in ferrets

**DOI:** 10.1128/mbio.02361-24

**Published:** 2025-01-08

**Authors:** Thomas Rowe, Ashley Fletcher, Melissa Lange, Yasuko Hatta, Gabriela Jasso, David E. Wentworth, Ted M. Ross

**Affiliations:** 1 Influenza Division, Centers for Disease Control and Prevention, Atlanta, Georgia, USA; 2 Department of Infectious Diseases, University of Georgia, Athens, Georgia, USA; 3 Ampersand Biosciences, Lake Clear, New York, USA; 4 Florida Research and Innovation Center, Cleveland Clinic, Port St. Lucie, Florida, USA; Max Planck Institute for Infection Biology, Berlin, Germany; Vaccine and Infectious Disease Organization (VIDO), Saskatoon, Saskatchewan, Canada

**Keywords:** influenza B, innate, adaptive, hemagglutinin inhibition, interferon, focus reduction assay, multiplex, ferret

## Abstract

Due to its natural influenza susceptibility, clinical signs, transmission, and similar sialic acid residue distribution, the ferret is the primary animal model for human influenza research. Antibodies generated following infection of ferrets with human influenza viruses are used in surveillance to detect antigenic drift and cross-reactivity with vaccine viruses and circulating strains. Inoculation of ferrets, with over 1,500 human clinical influenza isolates (1998–2019) resulted in lower antibody responses (HI <1:160) to 86% (387 out of 448) influenza B viruses (IBVs) compared to 2.7% (30 out of 1,094) influenza A viruses (IAVs). Here, we show that the immune responses in ferrets inoculated with IBV were delayed and reduced compared to IAV. Innate gene expression in the upper respiratory tract and blood indicated that IAV generated a strong inflammatory response, including an early activation of the interferon (IFN), whereas IBV elicited a delayed and reduced response. Serum levels of cytokines and IFNs were all much higher following IAV infection than IBV infection. Pro-inflammatory, IFN, TH1/TH2, and T-effector proteins were significantly higher in sera of IAV-infected than IBV-infected ferrets over 28 days following the challenge. Serum levels of Type-I/II/III IFNs were detected following IAV infection throughout this period, whereas Type-III IFN was only late for IBV. An early increase in IFN-lambda corresponded to gene expression following IAV infection. Reduced innate immune responses following IBV infection reflected the subsequent delayed and reduced serum antibodies. These findings may help in understanding the antibody responses in humans following influenza vaccination or infection and consideration of potential addition of innate immunomodulators to overcome low responses.

**IMPORTANCE:**

The ferret is the primary animal model for human influenza research. Using a ferret model, we studied the differences in both innate and adaptive immune responses following infection with influenza A and B viruses (IAV and IBV). Antibodies generated following infection of ferrets is used for surveillance assays to detect antigenic drift and cross-reactivity with vaccine viruses and circulating influenza strains. IAV infection of ferrets to generate these reagents resulted in a strong antibody response, but IBV infection generated weak antibody responses. In this study using influenza-infected ferrets, we found that IAV resulted in an early activation of the interferon (IFN) and pro-inflammatory response, whereas IBV showed a delay and reduction in these responses. Serum levels of IFNs and other cytokines or chemokines were much higher in ferrets following IAV infection. These reduced innate responses were reflected the subsequent delayed and reduced antibody responses to IBV in the sera. These findings may help in understanding low antibody responses in humans following influenza B vaccination and infection and may warrant the use of innate immunomodulators to overcome these weak responses.

## INTRODUCTION

Influenza A and B viruses (IAV and IBV) circulate globally and represent a major public health concern. Prevention from infection or severe illness relies on a robust adaptive immune response. This study involves the use of ferrets to better understand why IBVs generate weak immune responses following infection. Ferrets are an ideal model to explore immune responses to influenza viruses because they are naturally susceptible to influenza including human influenza strains without the need for prior adaptation (1, 2). Moreover, ferrets exhibit similar lung physiology and distribution of α2,3- and α2,6-linked sialic acids (3, 4), which are the receptors for influenza binding to cells.

Low antibody responses in humans infected with IBVs have been observed (5). Victoria lineage (B-Vic) and Yamagata lineage (B-Yam) infections showed that <50% of PCR-confirmed individuals had detectable neutralizing antibody titers. The effectiveness of IBV vaccine was also shown to be low, especially in children (6–9). Since low antibody responses are seen in humans infected or vaccinated with IBV, and ferrets inoculated with IBVs generate much lower immune responses compared to infection with seasonal IAVs, further investigation is required to identify ways to improve the immune responses to IBV.

Following exposure to influenza virus, the innate immune system is activated by interaction with pattern recognition receptors (PRRs) on susceptible cells. There are three different PRRs that sense influenza virus, which include retinoic acid-inducible gene I (RIG-I), Toll-like receptors (TLR3, TLR7, and TLR8) and the nucleotide-binding oligomerization domain (NOD)-like receptors (10). Pro-inflammatory cytokines and type-I interferons (IFNs) are induced through RIG-I and TLR7 pathways (11). At the site of infection (respiratory epithelial cells), TLR3 and TLR7 sense viral double-stranded and single-stranded RNA (ssRNA), respectively, within the endosome, while RIG-I recognizes cytosolic ssRNA or viral RNA containing 5′-triphosphate and induces Type I and III IFN responses through the transcriptional factors NF-kB and interferon regulatory factor (IRF) by interacting with mitochondrial antiviral signaling (MAVS) protein. These interactions result in the activation of NF-kB and IRF3 which result in initiating a pro-inflammatory response by the production of cytokines and chemokines as well as a Type-I/III IFN response in respiratory epithelial cells. In IFN-knock out studies in mice, interferon-beta (IFNB) has been shown to play an important role in defense against IAV in lung epithelial cells, and IFNB absence results in delayed expression of interferon-alpha (IFNA); thus, increasing the probability that the infecting virus can overrun the innate immune response of the host (12). The pro-inflammatory cytokines and chemokines can activate resident immune cells (innate lymphoid cells, macrophages, and dendritic cells [DCs]) and recruit cells to the site of infection. Additionally, cytokines and chemokines can stimulate a T-helper 1 (TH1) response by interferon-gamma (IFNG) and interleukin (IL)-12 (IL-12). They can also stimulate a T-effector response activation of signal transducer and activation of transcription (STAT) 3 (STAT3) resulting in IL-6 and IL-23 secretion to further upregulate the inflammatory response (13). These cytokines bind to the epithelial cell IFNA receptors (IFNARs) or those expressed by myeloid cells causing the expression of interferon stimulating genes (ISGs) and an antiviral state. In parallel, cytosolic ssRNA and DAMPs (Danger-Associated Molecular Patterns) can also interact with NOD-, LRR-, and pyrin domain-containing protein 3 (NLRP3) of the inflammasome complex to cause cleavage and activation of caspase-1 and induction of IL-1b and IL-18. The regulatory effects on adaptive immunity are not a result of direct action of Type-III IFN (IFNL) but rather indirectly through thymic stromal lymphopoietin (TSLP). IFNL secreted by infected respiratory epithelial cells can induce M cells to release TSLP which acts on migrating CD103^+^ DCs to stimulate the adaptive immune responses by enhancing CD8^+^ T cell maturation and promoting germinal center reactions in the lymph node through T-follicular/helper (Tfh) cells (14). Tfh cells help in B-cell survival and proliferation in germinal centers (14–16) resulting in an increase of IgG1 and IgA antibodies (16). Once these receptors are triggered by the virus, activation of several antiviral signals and production of cytokines and chemokines occurs to initiate the immune response in the host.

The humoral immune response plays a major role in immunity to influenza. This study focuses on the antibody response to influenza hemagglutinin (HA) which is the most commonly measured correlate of protection (17, 18). Cytokine and chemokine levels in serum have been shown to correlate with increased antibody responses following vaccination and infection. IL-2 can work together with cyclic adenosine 3′,5′-phosphate in synergy to enhance the antibody production by B-cells (19). In elderly human subjects, IL-2 treatment enhanced antibody responses to influenza vaccination (20). IL-2 has been shown to play an essential role in a T-helper 2 (TH2) differentiation (21) and may play a role in the adaptive immune response (22). Inflammatory cytokine levels of IL-6 and TNFA in serum have shown a positive correlation with fold-increases in hemagglutination inhibition (HI) titers following vaccination (23). IFN levels have also been shown to be protective and enhance antibody response following influenza infection. In mice lacking Tfh cells, which are important in humoral immunity, TH1 cells are the source of IFNG that promotes a protective IgG2 antibody response (24). IFNL has also been shown to increase adaptive mucosal antibodies (16). A systems biology approach in humans has shown that following influenza vaccination, strong innate immune responses, including early expression of IFN could predict high versus low antibody responses to HA (25, 26). In a study comparing influenza vaccination in the elderly to young adults, reduced B cell responses were observed in the elderly following influenza vaccination. Transcriptomic profiling revealed potential regulators of vaccine immunity indicating an early impairment of the innate immune responses in the elderly, which included both antiviral and IFN-related genes (27). A strong early innate response, marked by the expression of IFNs and activation of DCs showed a positive association with later antibody responses.

This study utilizes newly developed ferret-specific cytokines/chemokines and genes to measure immune responses and discern different responses to IAV and IBV. Taken together, this study explores innate and cytokine responses following IAV and IBV infection of ferrets and their contribution to development of robust adaptive antibody responses. Understanding factors which affect the ability of influenza viruses to generate robust immune responses may aid in the testing and improvement of influenza B vaccine efficacy in humans.

## MATERIALS AND METHODS

### Viruses

All influenza viruses were passaged in either MDCK “C#” or MDCK-SIAT1 ”S#” (28) cells according to established procedures (29, 30). Representative IAVs used in this study were: A/California/07/2009 “CA” (H1PDM09 subtype; passage C3) and A/Kansas/14/2017 “KS” (H3N2 subtype; passage S3). Representative IBVs used in this study were: B/Brisbane/60/2008 “BR” (B-Victoria lineage; passage CXC6) and B/Phuket/3073/2013 “PH” (B-Yamagata lineage; passage C4). Viral titers were determined by focus forming assay (FFA) (31) and given as log Focus Forming Units per milliliter (FFU/mL). Virus stocks were: CA (10^6.64^ FFU/mL), KS (10^7.00^ FFU/mL), BR (10^7.17^ FFU/mL), and PH (10^7.54^ FFU/mL).

### Ferret infection with influenza viruses and monitoring

All animal procedures were approved by the Institutional Animal Care and Use Committee in an Association for Assessment and Accreditation of Laboratory Animal Care International-accredited facility (IACUC Protocol: 3182ROWFERC). Male ferrets (Triple F Farms, Sayre, PA), aged 10–18 months and seronegative for circulating influenza viruses were used. Experiments were conducted using 18 ferrets for each virus [CA (A/H1PDM09), KS (A/H3N2), BR (B/Victoria lineage), and PH (B/Yamagata lineage)]. Anesthetized ferrets were challenged intranasally with 4 × 10^5^ FFU virus. Animals were monitored for 28 days for weight loss, fever, lethargy, sneezing, and dyspnea. Ferrets were weighed on Day 0 prior to challenge to establish a baseline and then daily for the first 10 days post-challenge and weekly thereafter through Day 28. Changes in weight were calculated as percentage loss or gain from Day 0. For fever calculation, a temperature transponder (IPTT-300; Bio Medic Data Systems [BMDS], Waterford, WI, USA) was implanted subcutaneously between the shoulder blades of each animal and read with a scanner (DAS-6007 IPTT Scanner System, BMDS). A baseline temperature (°F) was determined by the average temperature over 3 days prior to challenge. Temperatures were assessed daily for 10 days post-challenge, then weekly and fever was determined as any temperature greater or equal to 2°F above baseline. Ferrets were assessed daily for clinical signs. Lethargy was determined by the relative inactivity index (RII) from Day 0 through Day 7 post-challenge; this period allowed for lethargy calculation throughout the period of active replication in the ferret respiratory tract. The scoring system for activity assessment was calculated as follows: 0, alert and playful; 1, alert but playful only when stimulated; 2, alert but not playful when stimulated; and 3, neither alert nor playful when stimulated. Based on the daily scores for each animal in a group, a relative inactivity index was calculated as follows: Σ_(Day 1 to Day 7)_ [score +1 ]_*n*_ / Σ_(Day 1 to Day 7)_*n*, where *n* equals the total number of observations. A value of 1 was added to each base score so that a score of 0 could be divided by a denominator, resulting in an index value of 1.0. RII, sneezing and dyspnea were assessed each day prior to handling and sedation of the animals. Clinical signs assessment was consistent with previously established methods (32, 33).

### Ferret sample collection and processing

Pre-challenge and on Days 1, 3, 5, 7, 10, 14, 21, and 28 post-challenge, nasal wash (NW) and blood samples were collected from ferrets for virologic, genetic, and antibody analyses. Animals were sedated followed by flushing the nares by instillation of 2 mL NW solution (phosphate-buffered saline [PBS], 1% bovine serum albumin [BSA], and antibiotics), inside a class-II biosafety cabinet, and collected when expelled in a Petri dish. NW was centrifuged (5 min × 5,000 rpm at 4°C), supernatant was stored at −80°C for virus titration. About 100 µL PBS was added to NW pellets followed by 280 µL AVL lysis buffer (Qiagen, Germantown, MD, USA). About 2–3 mL blood was collected in an SST tube for serum separation. Serum was tested for antibodies to influenza virus and cytokines.

On Days 1, 3, 5, 7, 10, and 28 post-challenge, three ferrets were euthanized for collection of sera, and cells. These animals were first anesthetized and blood was collected in SST tubes (serum) and K2/EDTA (cell purification). Serum was separated from SST tubes by centrifugation for 10 min at 1,500 × *g* at room temperature. Peripheral blood mononuclear cells (PBMCs) were isolated from blood collected in K2/EDTA tubes and overlayed onto Histopaque-1077 (Sigma, St. Louis, MO, USA). Cells obtained from the interface were treated with ammonium chloride to lyse any remaining red blood cells. The final cell pellet was resuspended in 1 mL cell culture medium. About 280 µL of AVL lysis buffer were added to 200 µl PBMC. Samples were mixed three to four times and lysate was frozen at −80°C until RNA extraction. Carrier RNA was added to each sample and RNA was extracted using EZ1 DSP kit on a Qiagen EZ1 Advanced XL extractor according to the manufacturer’s instructions (Qiagen, Germantown, MD, USA). RNA was eluted in 120 µl RNase-free water and stored at −80°C until evaluated for gene expression by qRT-PCR. Blood was collected from an additional three mock-infected ferrets and included as naïve controls.

### Virus kinetics by FFA

NW samples collected from influenza virus-infected ferrets were tested for virus with a FFA as previously described (31). Briefly, samples were serially diluted in virus growth medium plus trypsin (DMEM, 0.1% BSA, 1 µg/mL TPCK-treated trypsin (Sigma, St. Louis, MO, USA)) and added to MDCK-SIAT1 cell monolayers (28) in 96-well flat-bottom plates in quadruplicate. Following a 2-h incubation at 37°C, an overlay containing Avicel RC/CL (34) (Type: RC581 NF; FMC Health and Nutrition, Philadelphia, PA, USA), 0.1% BSA, antibiotics, and TPCK-treated trypsin was added. Plates were incubated overnight at 37**°**C, 5% CO_2_, fixed, permeabilized, and stained with a monoclonal antibody pool to influenza A or B nucleoprotein (International Reagent Resource; https://www.internationalreagentresource.org/). Infectious foci were visualized using TrueBlue substrate (Sera Care, Inc., Milford, MA, USA) and enumerated using a CTL Bio Spot Analyser with ImmunoCapture 6.4.87 software (Cellular Technology Ltd., Shaker Heights, OH, USA). FFA titer was determined by multiplying sample dilution which gave between 100 and 300 spots by the spot number at that dilution, to obtain the FFU/mL. The foci in the cell control were subtracted and the number of foci remaining was multiplied by 20 to give FFU/mL. The limit of detection was 10^2.3^ FFU/mL.

### Antibody detection by HI assay

Ferret sera were treated with receptor-destroying enzyme (RDE) (Denka Co Ltd, Tokyo, Japan) and adsorbed with turkey erythrocytes (TRBC), and tested by HI assay according to established procedures (29). RDE-treated and adsorbed sera were twofold serially diluted in v-bottom 96-well plates followed by the addition of 4 hemagglutination units of influenza virus (CA, KS, BR, or PH), followed by the addition of 0.5% TRBC and mixed. The HI titers were determined as the reciprocal of the last serum dilution which inhibited the hemagglutination of the TRBCs by the virus.

### Neutralizing antibody level detection by focus reduction assay

The focus reduction assay (FRA), initially developed by the WHO Collaborating Centre in London, United Kingdom, was modified and utilized in this study. MDCK-SIAT1 cells were seeded in 96-well plates in Dulbecco’s modified Eagle medium (DMEM) containing 5% heat-inactivated FBS and antibiotics. The following day, the confluent cell monolayers were rinsed with 0.01 M PBS at pH 7.2, (Gibco BRL, ThermoFisher Scientific Inc., Waltham, MA, USA) followed by the addition of twofold serially diluted RDE-treated ferret sera at 50 µL per well starting with 1:20 dilution in virus growth medium containing 1 µg/mL TPCK-treated trypsin, VGM-T (DMEM, 0.1% fraction-V BSA, antibiotics [penicillin/streptomycin], and 1 µg/mL TPCK-treated trypsin). Afterwards, 50 µL standardized virus in VGM-T was added to each plate or VGM-T to cell control wells. The virus stocks were standardized by FFA to determine FFU/mL. Following a 2-h incubation period at 37**°**C, a 100 µL overlay consisting of equal volumes of 1.2% Avicel RC/CL (34) in 2× MEM containing 1 µg/mL TPCK-treated trypsin, 0.1% BSA, and antibiotics was added to each well. Plates were incubated for 18–22 h at 37**°**C, 5% CO_2_. The overlays were removed from each well and the monolayer was washed once with PBS to remove any residual Avicel. The plates were fixed with ice-cold 4% (wt/vol) paraformaldehyde in PBS (10% formalin) for 30 minutes at 4**°**C, followed by a PBS wash and cell permeabilization using 0.5% Triton X-100 in PBS/glycine at room temperature for 20 min. Plates were washed three times with PSBT (PBS, 0.1% Tween-20), incubated for 1 h with a monoclonal antibody pool against influenza A or B nucleoprotein (35) in ELISA buffer (PBS,10% horse serum, and 0.1% Tween-80). Following three washes with PBST, the cells were incubated with goat anti-mouse peroxidase-labeled IgG (Sera Care, Inc., Milford, MA, USA) in ELISA buffer for 1 h at room temperature. Plates were washed three times with PBST, and infectious foci (spots) were visualized using TrueBlue substrate containing 0.03% H_2_O_2_ and incubated at room temperature for 10–15 min. The reaction was stopped by washing five times with deionized water. Plates were dried and the foci were enumerated using a CTL Bio Spot Analyzer with ImmunoCapture 6.4.87 software (Cellular Technology Ltd., Shaker Heights, OH, USA). The FRA titer was reported as the reciprocal of the highest dilution of serum corresponding to 50% foci reduction compared to the virus control (VC) minus the cell control (CC).

### Gene expression in ferret cells

Ferret primers generated to pro-inflammatory (*MCP1*, *IL-1B*, and *IL-6),* TH1 (*CXCL10*), TH2 (*IL-2*), T-regulatory (*TGFB1*), T-effector (*IL-4*, *IL-12p40*, and *IL-17*), apoptosis (*Granzyme A*), Type-I/II/III IFNs (*IFNA*, *IFNB*, *IFNG*, and *IFNL3*), IFN responses (*STAT1*, *STAT2*, *STAT3*, *RIG-I*, *SOCS3*, and *TSLP*) and housekeeping (*GAPDH*) genes were used. Genes using TaqMan, probes were modified with 6-FAM, fluorescein amidites (FAM) fluorophore on the 5′ end and a non-fluorescent Black Hole Quencher-1 (BHQ-1) on the 3′ end. Additionally, a locked nucleic acid at adenine <LNA A> was incorporated in all probes in order to increase template binding strength for real-time PCR (36). Primers and probes for all ferret genes were generated from published ferret sequences (31, 37–39). All primer/probe sets used are shown in Table S1.

Quantitative real-time PCR (qRT-PCR) was performed using an ABI 7500 Fast Dx Real-Time PCR instrument (Applied Biosystems, Waltham, MA, USA). PCR reactions were performed in a 5 µl RNA reaction volume using SYBR Green qPCR SuperMix (Applied Biosystems) or SuperScript III Platinum One-Step qRT-PCR Kit for TaqMan reactions (InvivoGen, San Diego, CA, USA). An RT reaction for 30 min at 50°C, inactivation for 2 min at 95°C, followed by 40 amplification cycles at an annealing temperature of 50°C. Reactions were performed on three ferrets for each virus and timepoint and the values were normalized by subtracting the mean value of the cycle threshold (*C*_*T*_) from that of the *C*_*T*_ for glyceraldehyde-3-phosphate dehydrogenase (GAPDH) housekeeping gene (Δ*C*_*T*_). The relative levels of gene expression for infected cells were determined by subtracting the individual Δ*C*_*T*_ values from that of average Δ*C*_*T*_ values of pre-infection cells (ΔΔ*C*_*T*_) and expressing the final quantification values (2^−ΔΔCT^) as relative fold changes. Genes upregulated >10^4^-fold (>10,000) were given a value of 10,000 and genes downregulated <10^−4^-fold (<0.0001) were given a value of 0.0001.

### Cytokine/chemokine levels in ferret sera by multiplex assay

Ferret serum samples were stored at −80°C until evaluated. Sera was tested by a Luminex ferret multiplex assay kit (Ampersand Biosciences [www.ampersandbio.com] Lake Clear, NY, USA] contained ferret-specific antibodies and proteins in a microsphere-based assay and consisted of antigen-specific antibodies covalently coupled to magnetic Luminex beads and biotinylated detection antibodies in a capture-sandwich format. Incubations were performed at room temperature in 96-well plates. About 30 µL of standard, controls, or serum samples (1:5 dilution) were added followed by 10 µL of multiplexed capture-antibody microspheres and 10 µL of blocking buffer to each well. The plates were sealed and incubated for 2 h on a shaker. The plates were washed three times followed by the addition of 40 µL of biotinylated detection antibody and incubated for 1 h on a shaker. About 20 μL streptavidin-phycoerythrin was added and incubated for 30 min. After washing, beads in the plates were resuspended in 100 µL of wash buffer, shaken for 5 min and analyzed on a Luminex 200 Analyzer (Luminex Corporation [www.luminexcorp.com)] Austin, TX, USA). Results were determined by extrapolating the analyte concentration from the mean fluorescence intensity (MFI) value using the standard curve. Results were generated and extracted using RBM Plate Reader and Plate Viewer analysis software, respectively. Data were exported with values represented by two significant figures and analyzed in Microsoft Excel and GraphPad Prism 10 (GraphPad Software, La Jolla, CA, USA).

### Type-III IFN bioassay

IFNL levels in influenza-infected ferret serum were detected using HEK-λ reporter cells (HEK-Blue IFN-λ cells: InvivoGen, San Diego, CA, USA) designed to monitor the activation of the JAK/STAT/ISGF3 pathway induced by Type III IFNs. The IFNL levels were measured by a colorimetric assay at 650 nm using Quanti-Blue solution according to the manufacturer’s instructions (InvivoGen, San Diego, CA, USA).

About 20 µL of ferret sera was added in triplicate wells of a 96-well tissue culture plate. HEK-λ cells were adjusted to a concentration of 2.8 × 10^5^ cells/mL. And 180 µL of HEK-λ cells was added to each well containing 20 µL of sample and to serially 1/2-log diluted (0.1–1,000 ng/ml) recombinant ferret IFNL3 (Kingfisher Biotech, St. Paul, MN, USA). The plates were incubated at 37°C, 5%CO_2_. After 20 h, 20 µL supernatant was transferred to a new 96-well plate and 180 µL of Quanti-Blue substrate was added and incubated for 2 h at 37°C, 5%CO_2_. Absorbance was read at 655 nm. A sigmoidal 4-point standard curve from 0.1 to 1,000 ng/mL was generated using recombinant ferret IFNL3 protein (Kingfisher Biotech [www.kingfisherbiotech.com], St. Paul, MN, USA) and unknown samples were extrapolated from the standard curve.

### Statistical analysis

GraphPad Prism 10 was used for all statistical analyses (GraphPad Software, La Jolla, CA, USA). One-way ANOVA was used to determine differences over time between viruses and significance between groups was determined by two-way ANOVA analysis. Gene expression analysis was performed from three ferrets per time point. Spearman (one-tailed, 95% confidence) correlation method was used for comparison of virus replication to gene expression or protein concentration (multiplex or bioassay) as well as antibody levels (HI or FRA) and protein concentration. The Student’s *t* test (unpaired) was used to assess the statistical differences in the gene expression levels with respect to the uninfected controls and for comparing IAV to IBV and antibody-secreting cells by ELISpot. A *P* value of <0.05 was considered statistically significant: *P < 0.05, **P < 0.01, ***P < 0.001, and ****P < 0.0001.

## RESULTS

### IAV virus replication kinetics differ from IBV and cause greater morbidity in ferrets

Ferrets were inoculated with representative influenza A/H1N1pdm09 (A/California/07/2009, “CA”), A/H3N2 (A/Kansas/14/2017, “KS”), B-Victoria lineage (B/Brisbane/60/2008, “BR”), or B-Yamagata lineage (B/Phuket/3073/2013, “PH”) virus. Throughout the challenge, NW and blood samples were collected for virus titration/molecular analyses and molecular/immunological analyses, respectively.

**Fig 1:**
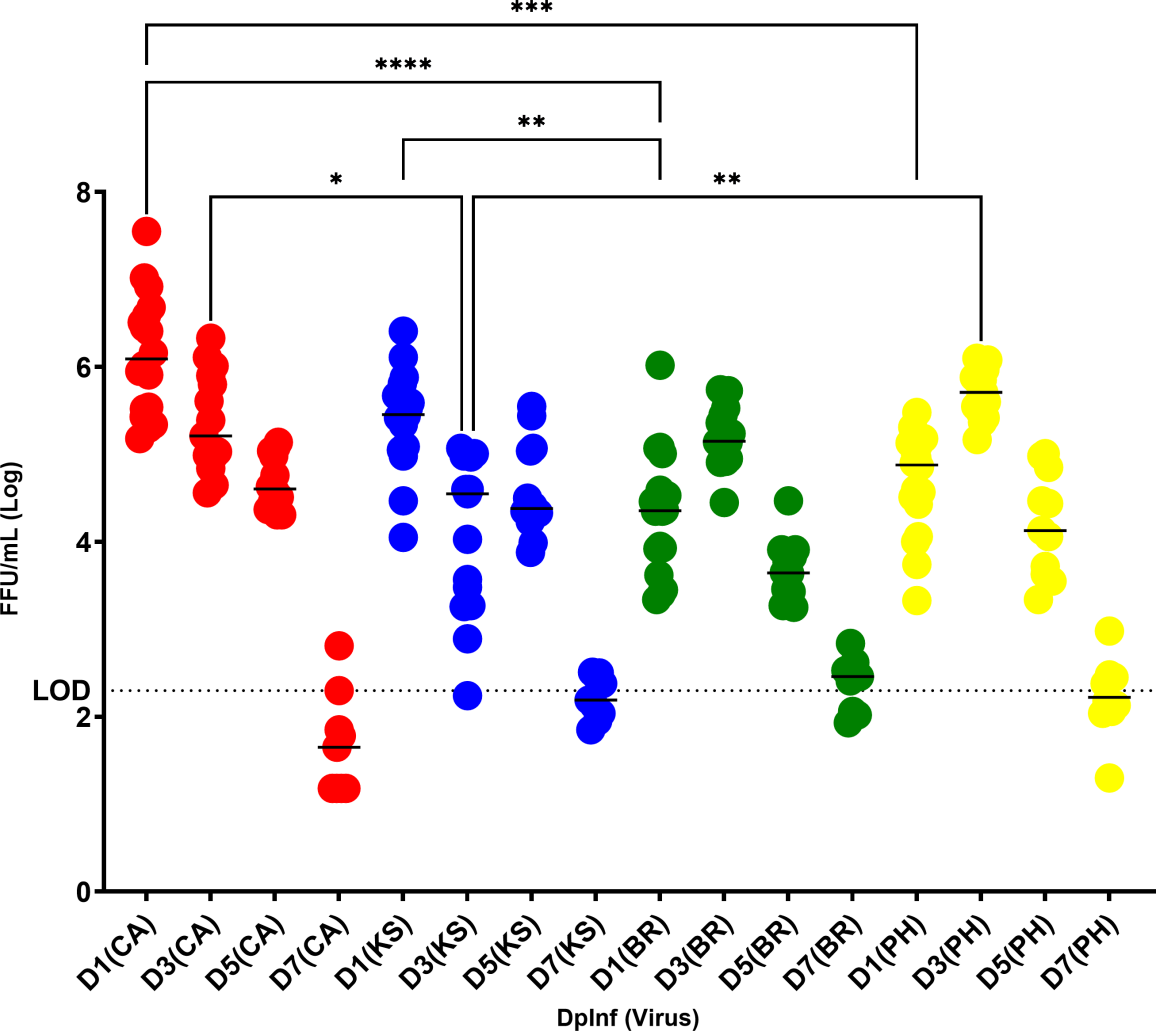
Replication kinetics of IAV [H1N1pdm09 “CA” (A/California/07/2009), H3N2 “KS” (A/Kansas/14/2017)] and IBV [B-Victoria lineage “BR” (B/Brisbane/60/2008), B-Yamagata lineage “PH” (B/Phuket/3073/2013)] in ferret upper respiratory tract over 7 days. Ferret numbers analyzed per time point post-challenge were: D1 and D3 (*n* = 18); D5 and D7 (*n* = 15). H1N1pdm09 = red, H3N2 = blue, B-Victoria = green, B-Yamagata = yellow. Limit of detection, indicated by a dotted line, was 10^2.3^ FFU/mL. Significance between groups was determined using one-way ANOVA (Sidak’s multiple comparison test).

Virus replication kinetics demonstrated peak early replication in the upper respiratory tract (URT) at D1 post-challenge in IAV-challenged animals and later replication at D3 in IBV-challenged animals (Fig. 1). IAV viral replication peaked earlier on D1 post-challenge (CA = 10^6.14^ FFU/mL, KS = 10^5.36^ FFU/mL), whereas IBV virus replication peaked later at D3 post-challenge (BR = 10^5.19^ FFU/mL, PH = 10^5.70^ FFU/mL) in ferret URT. There was a significant difference between IAV and IBV on D1 (CA vs BR [*P* < 0.0001] and vs PH [*P* = 0.007]; KS vs BR [*P* = 0.002]); however, by D3 IBV replication increased to equivalent levels. PH, however, replicated to higher levels than KS on D3 (*P* = 0.003). CA replicated significantly higher and earlier than IBV (BR, *P* < 0.0001; PH, *P* = 0.0236) as well as KS (*P* = 0.0015). These replication kinetics confirm greater morbidity of CA in ferrets compared to other viruses tested (Table 1) and compliment previous findings of this virus compared to other seasonal influenza viruses (40).

**TABLE 1:** Ferret clinical signs following infection with influenza A and B viruses^*a*^

Virus (type)	Inactivity^*b*^ (D1–D7pInf)	Days sneezing (%)	% Weight loss^*c*^ (day)	Elevated temperature^*d*^ (days)	NW viral load^*e*^ (avg ± SD log_10_ FFU/mL)	
					D1	D3
CA (A/H1N1pdm09)	1.37	D4–D5 (25%)	7.6% (D6)	+2.3°F (D2)	**6.14 ± 0.68**	5.36 ± 0.56
KS (A/H3N2)	1.26	D2 (80%)	4.3% (D3)	+2.7°F (D2,D4)	**5.41 ± 0.55**	4.10 ± 0.92
BR (B-Victoria)	1.04	D3 (27%)	2.7% (D3)	+2.7°F (D2)	4.33 ± 0.69	**5.19 ± 0.34**
PH (B-Yamagata)	1.01	D2 (40%)	4.9% (D3)	+2.4°F (D2)	4.69 ± 0.59	**5.70 ± 0.27**

^*a*^ Ferret numbers analyzed per time point post challenge were: D0 –D3 (*n* = 18); D4 – D7 (*n* = 15).

^*b*^ Relative inactivity index “RII” (D1–D7) post-challenge. RII = $\sum_{\left(D1-7\right)}[score+1]n/\sum_{\left(D1-7\right)}n$

^*c*^ Day post-challenge of peak weight loss percentage (day) from pre-challenge weight from D1 to D7 post-challenge.

^*d*^ Average elevated temperature of at least +2°F (fever) above baseline (days post-challenge elevated). Fever indicated by any temperature greater than the average baseline (D2 to D0 pre-challenge) +2°F.

^*e*^ Average viral load in ferret URT on D1 and D3 post-challenge. Peak viral load in bold. Baseline = 2.3 Log_10_ FFU/mL.

Following challenge with influenza viruses, ferrets exhibited classical clinical signs of infection including elevated temperature, weight loss, lethargy, and sneezing as well as virus replication in the URT (Table 1). Regardless of the virus used, all animals developed fever at least +2°F over pre-challenge temperature by D2 post-challenge with average temperature increases being similar between IAVs (+2.5°F) and IBVs (+2.55°F). No significant differences between groups were seen (two-way ANOVA). All animals demonstrated weight loss through D7 post-challenge. The maximum weight loss from pre-challenge weight occurred on D3 for IBV (BR [−2.7%], PH [−4.9%]) and IAV (KS [−4.3%]); however, CA peak weight loss occurred later on D6 (−7.6%). Significant weight loss was seen with IAV (CA [*P* < 0.0001], KS [*P* = 0.049]) compared to IBV (BR) over the first 10 days following challenge (two-way ANOVA). Over the same period, PH weight loss was significantly greater than BR as well (*P* < 0.0001). Daily temperature changes and weight loss can be found in Fig. S1. For activity following challenge, only IAVs showed marked lethargy compared to IBVs ranging from IAV CA having the highest relative inactivity index (RII = 1.37) followed by KS (RII = 1.26) then the IBVs showing minimal lethargy BR (RII = 1.04) and PH (RII = 1.01). Taken together, clinical signs indicate that these IAVs cause greater morbidity in ferrets than the IBVs tested.

### IAV induces earlier inflammatory and IFN gene expression than IBV in ferret respiratory tract and peripheral blood

To determine the temporal relationship between innate immune responses and development of antibodies following challenge with influenza virus in ferrets, we conducted a comparison of host gene expression in NW cells in the URT and PBMC in the peripheral blood using qRT-PCR (Tables 2 and 3). Upregulated gene expression at the site of infection in the URT (Table 2) shows that of the nine upregulated genes (gene expression >1 over mock challenged) on D3 post-challenge (*MCP-1*, *CXCL10*, *TGFB1*, *STAT1*, *STAT2*, *STAT3*, *RIG-I*, *SOCS3*, and *TSLP*), seven genes (*MCP-1*, *CXCL10*, *STAT1*, *STAT2*, *STAT3*, *RIG-I*, and *SOCS3*) showed greatest fold-increase following IAV (either CA or KS) infection, whereas only two genes (*TGFB1* and *TSLP*) were highest following IBV (PH) infection. By D5 post-challenge four additional genes (*IL-2*, *IL-4*, *IFNG*, and *IFNL3*) were only upregulated following IAV challenge (CA), indicating initiation of adaptive effector T cell and IFN response. All other genes upregulated on D3 continued to be upregulated on D5. IBV infection resulted in high continued upregulation (>50-fold) of *SOCS3* gene on Days 3 and 5 as well IAV (KS). Corresponding *IFNG* and *IFNL3* expression was suppressed for these samples indicating a potential inhibition of IFN signaling (41). Trends in gene responses were observed and inferences were made based on these responses; however, statistical significance was minimal due to small sample size.

**TABLE 2:** Comparison of ferret gene expression following IAV and IBV challenge in the URT and peripheral blood by qRT-PCR (by virus)^*a*^

		URT (NW)							PBMC						
Gene	DpInf	A/H1p CA	A/H3 KS	B/Vic BR	B/Yam PH	DpInf	Highest expressor^*b*^	Highest expression^*c*^	A/H1p CA	A/H3 KS	B/Vic BR	B/Yam PH	DpInf	Highest expressor^*b*^	Highest expression^*c*^
*MCP1*	D3	14.4	10.0	12.0	3.8	D3	CA	14.4	↓	↓	↓	↓	D3	-	-
	D5	227.3	2.5	4.9	10.7	D5	CA	227.3	↓	3.6	↓	↓	D5	KS	3.6
*CXCL10*	D3	28.1	25.6	24.3	21.7	D3	CA	28.1	1.4	1.2	1.7	2.1	D3	PH	2.1
	D5	10.4	24.6	25.1	21.9	D5	BR	25.1	1.3	1.9	1.7	1.5	D5	KS	1.9
*IL-2*	D3	↓	↓	↓	↓	D3	-	-	↓	↓	↓	↓	D3	-	-
	D5	19.7	↓	↓	↓	D5	CA	19.7	↓	↓	↓	↓	D5	-	-
*TGFB1*	D3	4.9	8.2	3.3	9.5	D3	PH	9.5	↓	↓	↓	↓	D3	-	-
	D5	330.3	2.9	3.0	3.0	D5	CA	330.3	↓	1.2	↓	↓	D5	KS	1.2
*IL-4*	D3	↓	↓	↓	↓	D3	-	-	↓	↓	↓	↓	D3	-	-
	D5	86.9	↓	↓	↓	D5	CA	86.9	↓	2.4	↓	↓	D5	KS	2.4
*IL-12p40*	D3	↓	↓	↓	↓	D3	-	-	↓	↓	↓	↓	D3	-	-
	D5	↓	↓	↓	↓	D5	-	-	↓	↓	↓	↓	D5	-	-
*IL-17*	D3	↓	↓	↓	↓	D3	-	-	↓	↓	↓	↓	D3	-	-
	D5	↓	↓	↓	↓	D5	-	-	↓	↓	↓	↓	D5	-	-
*IL-1B*	D3	↓	↓	↓	↓	D3	-	-	↓	↓	↓	↓	D3	-	-
	D5	↓	↓	↓	↓	D5	-	-	↓	↓	↓	↓	D5	-	-
*IL-6*	D3	↓	↓	↓	↓	D3	-	-	↓	↓	↓	↓	D3	-	-
	D5	↓	↓	↓	↓	D5	-	-	↓	↓	↓	↓	D5	-	-
*Granzyme A*	D3	↓	↓	↓	↓	D3	-	-	↓	↓	↓	↓	D3	-	-
	D5	↓	↓	↓	↓	D5	-	-	↓	↓	↓	↓	D5	-	-
*IFNA*	D3	↓	↓	↓	↓	D3	-	-	↓	↓	↓	↓	D3	-	-
	D5	↓	↓	↓	↓	D5	-	-	↓	↓	↓	↓	D5	-	-
*IFNB*	D3	↓	↓	↓	↓	D3	-	-	↓	↓	↓	↓	D3	-	-
	D5	↓	↓	↓	↓	D5	-	-	↓	↓	↓	↓	D5	-	-
*IFNG*	D3	↓	↓	↓	↓	D3	-	-	↓	↓	↓	↓	D3	-	-
	D5	1.6	↓	↓	↓	D5	CA	1.6	↓	↓	↓	↓	D5	-	-
*IFNL3*	D3	↓	↓	↓	↓	D3	-	-	↓	↓	↓	↓	D3	-	-
	D5	62.5	↓	↓	↓	D5	CA	62.5	↓	5.7	↓	↓	D5	KS	5.7
*STAT1*	D3	109.5	101.9	99.4	76.6	D3	CA	109.5	↓	↓	↓	↓	D3	-	-
	D5	142.7	92.6	98.9	94.7	D5	CA	142.7	↓	↓	↓	↓	D5	-	-
*STAT2*	D3	515.5	477.4	436.5	302.2	D3	CA	515.5	28.4	18.8	26.4	21.5	D3	CA	28.4
	D5	176.4	358.5	481.0	365.8	D5	BR	481.0	19.5	8.4	34.1	29.7	D5	BR	34.1
*STAT3*	D3	199.8	179.8	198.1	118.7	D3	CA	199.8	↓	1.5	1.4	1.4	D3	KS	1.5
	D5	75.9	154.9	178.0	182.0	D5	PH	182.0	↓	2.1	1.2	↓	D5	KS	2.1
*RIG-I*	D3	3144.0	2450.0	2235.0	1567.0	D3	CA	3144.0	43.3	16.4	36.6	11.2	D3	CA	43.3
	D5	624.5	2167.0	2333.0	1684.0	D5	BR	2333.0	50.2	7.1	51.8	51.9	D5	BR	51.9
*SOCS3*	D3	88.4	82.2	80.7	57.2	D3	CA	88.4	3.0	3.0	3.1	2.8	D3	BR	3.1
	D5	26.4	78.7	81.3	73.8	D5	BR	81.3	3.5	1.2	4.1	3.7	D5	BR	4.1
*TSLP*	D3	↓	↓	↓	1.4	D3	PH	1.4	↓	↓	↓	↓	D3	-	-
	D5	200.9	↓	↓	↓	D5	CA	200.9	↓	↓	↓	↓	D5	-	-

^*a*^ Comparison of gene expression by IAV (CA, KS) and IBV (BR, PH) in the URT (NW) and peripheral blood (PBMC) of ferrets on D3 and D5 post-challenge by qRT-PCR. Gene expression in the URT was determined in NW cells and peripheral blood expression was determined in PBMC. Average fold upregulated genes over pre-infection (D0) mock challenged animals for IAV (CA and KS) and IBV (BR and PH) are shown. An upregulation of expression (>1-fold) is shown. Downregulated genes are indicated by “↓”.

^*b*^ Virus which exhibited the greatest fold increase for each gene. - indicates not upregulated.

^*c*^ Fold expression values of highest expressor.

**TABLE 3:** Comparison of ferret gene expression following IAV and IBV challenge in the URT and peripheral blood by qRT-PCR (IAV and IBV comparison)^*a*^

Gene	DpInf	URT (NW)			PBMC		
		IAV	IBV	Highest expressor^*b*^	IAV	IBV	Highest expressor^*b*^
*MCP1*	D3	11.8	7.9	IAV	↓	↓	-
	D5	92.4	8.4	IAV	1.8	↓	IAV
*CXCL10*	D3	26.6	23.0	IAV	1.3	1.9	IBV
	D5	18.9	23.1	IBV	1.6	1.6	IAV/IBV
*IL-2*	D3	↓	↓	-	↓	↓	-
	D5	7.9	↓	IAV	↓	↓	-
*TGFB1*	D3	6.9	6.4	IAV	↓	↓	-
	D5	133.8	3.0	IAV	↓	↓	-
*IL-4*	D3	↓	↓		↓	↓	-
	D5	34.7	↓	IAV	1.3	↓	IAV
*IL-12p40*	D3	↓	↓		↓	↓	-
	D5	↓	↓		↓	↓	-
*IL-17*	D3	↓	↓		↓	↓	-
	D5	↓	↓		↓	↓	-
*IL-1B*	D3	↓	↓		↓	↓	-
	D5	↓	↓		↓	↓	-
*IL-6*	D3	↓	↓		↓	↓	-
	D5	↓	↓		↓	↓	-
*Granzyme A*	D3	↓	↓		↓	↓	-
	D5	↓	↓		↓	↓	-
*IFNA*	D3	↓	↓		↓	↓	-
	D5	↓	↓		↓	↓	-
*IFNB*	D3	↓	↓		↓	↓	-
	D5	↓	↓		↓	↓	-
*IFNG*	D3	↓	↓		↓	↓	-
	D5	↓	↓		↓	↓	-
*IFNL3*	D3	↓	↓		↓	↓	-
	D5	25.0	↓	IAV	2.8	↓	IAV
*STAT1*	D3	105.0	88.0	IAV	↓	↓	-
	D5	112.7	96.4	IAV	↓	↓	-
*STAT2*	D3	492.6	369.4	IAV	23.6	23.9	IBV
	D5	285.6	411.9	IBV	13.9	31.9	IBV
*STAT3*	D3	187.8	158.4	IAV	1.2	1.4	IBV
	D5	123.3	180.4	IBV	1.5	↓	IAV
*RIG-I*	D3	2,727.6	1,900.8	IAV	29.8	23.9	IAV
	D5	1,550.3	1,943.7	IBV	28.7	51.8	IBV
*SOCS3*	D3	84.7	68.9	IAV	3.0	2.9	IAV
	D5	57.7	76.8	IBV	2.3	3.9	IBV
*TSLP*	D3	↓	↓		↓	↓	-
	D5	80.7	↓	IAV	↓	↓	-

^*a*^ Comparison of gene expression by IAV and IBV in the URT (NW) and peripheral blood (PBMC) of ferrets on D3 and D5 post-challenge by qRT-PCR. Gene expression in the URT was determined in NW cells and peripheral blood expression was determined in PBMC. Comparison of upregulated genes for IAV and IBV on D3 and D5 post-challenge. Average gene expression. Gene down-regulated = “↓”.

^*b*^ Virus type, IAV or IBV, with greatest fold increase for each gene. Virus type indicated where fold increase is >1, otherwise “-”.

Gene expression in the peripheral blood (Table 2) showed fewer upregulated genes compared to the URT, on both D3 and D5 post-challenge. *IL-2*, *IFNG*, *STAT1,* and *TSLP* genes were not upregulated in PBMC following IAV or IBV infection. On D3 post-challenge, only five genes were upregulated (*CXCL10*, *STAT2*, *STAT3*, *RIG-I*, and *SOCS3*) with the greatest fold-increase following IBV infection. Three of these genes (*STAT2*, *STAT3*, and *RIG-I*) showed greater upregulation following IAV infection; whereas two genes (*CXCL10* and *SOCS3*) were upregulated to a greater extent following IBV infection. By D5 post-challenge, four additional genes were upregulated (*MCP-1*, *TGFB1*, *IL-4*, and *IFNL3*). All of these genes were upregulated only following IAV infection. By comparing gene upregulation in IAV to IBV (Table 3), all upregulated genes were the highest following IAV infection early (D3) in the URT followed by a switch to IBV of *CXCL10*, *STAT2*, *STAT3*, *RIG-I*, and *SOCS3* by D5. Only *IL-4* and *IFNL3* were upregulated in PBMC following IAV infection. Both gene expression increases and decreases for all tested genes, including significant differences between viruses, are shown in Table S2 for URT (A) and PBMC (B), respectively. These gene expression levels indicate that IAV resulted in an earlier innate immune response in both the URT and peripheral blood.

### Serum cytokine and chemokine levels are elevated following IAV infection in ferrets

**Fig 2:**
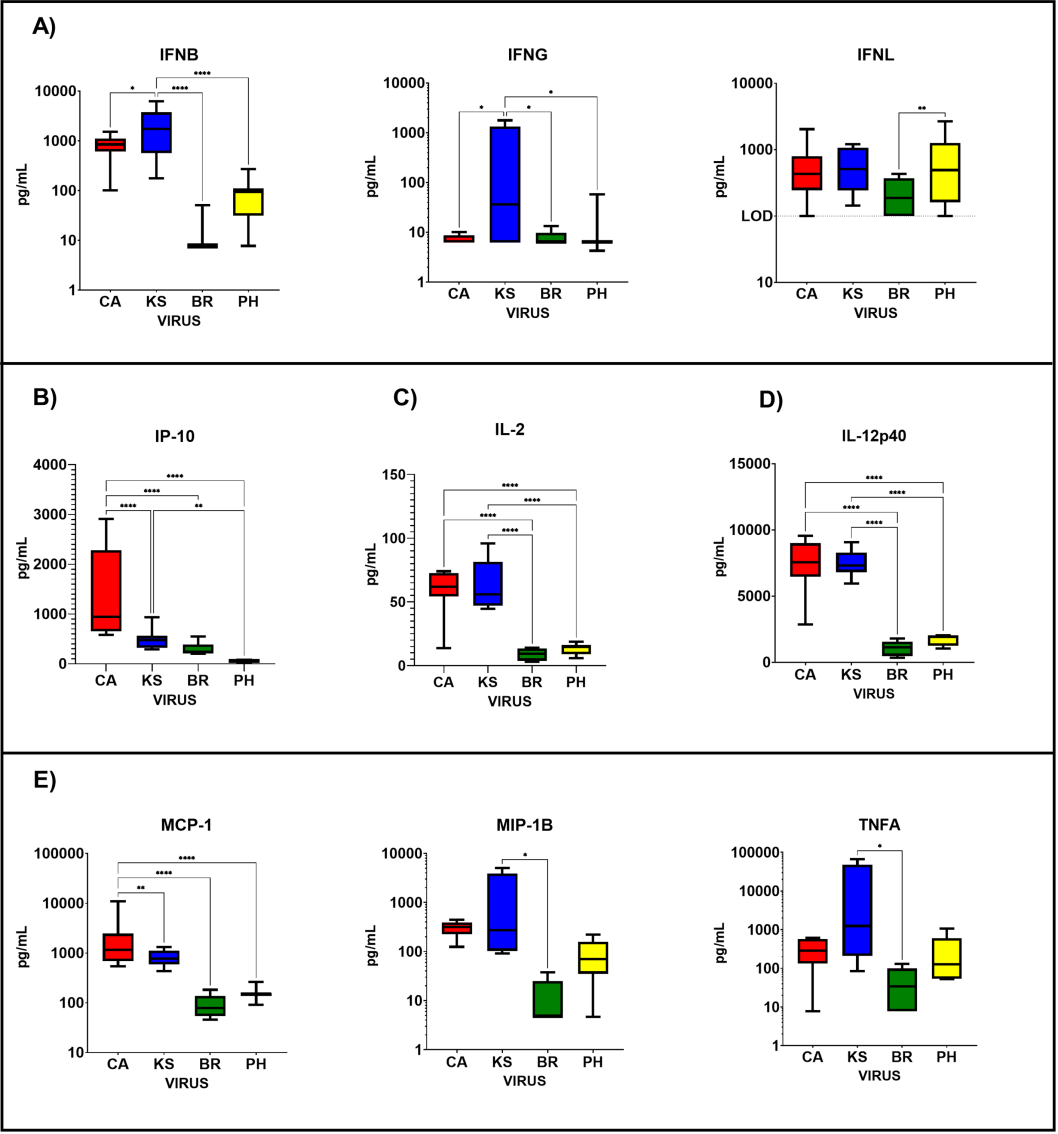
Cytokine and chemokine levels in ferret sera following IAV or IBV infection. Cytokine and chemokine levels in sera following IAV (CA and KS) or IBV (BR and PH) infection (Days 1–28) in ferrets (*N* = 3/time point). Time points post infection include: D0, D1, D3, D5, D7, D10, D14, D21, and D28. Influenza A viruses: H1N1pdm09 A/California/07/2009, “CA” (red) and H3N2 A/Kansas/14/2017, “KS” (blue). Influenza B viruses: B-Victoria lineage B/Brisbane/60/2008, “BR” (Green) and B-Yamagata lineage B/Phuket/3073/2013, “PH” (yellow). (**A**) Type-I (IFNB), Type-II (IFNG), and Type-III (IFNL) interferons. (**B**) TH1 chemokine (IP-10). (**C**) TH2 cytokine (IL-2). (**D**) T-effector cytokine (IL-12p40). (**E**) Pro-inflammatory chemokines (MCP-1 and MIP-1B) and cytokine (TNFA). Two-way ANOVA used to determine significant differences between viruses over a 28-day period.

Serum cytokine and chemokine levels in ferrets following challenge with IAV and IBV were evaluated over a 1-month period. Ferret sera were tested using a multiplex assay for ferret analytes (IFNB, IFNG, IP-10, IL-2, MCP-1, MIP-1B, IL-4, IL-17, IL-12p40, IL-12p70, TNFA, IL-6, and IL-8) and by bioassay (IFNL). Significant differences (two-way ANOVA) between IAV (CA and KS) and IBV (BR and PH) were observed for several analytes (Fig. 2). Type-I (IFNB) and Type-II (IFNG) interferons showed significantly higher levels in ferret sera following challenge with IAV compared to IBV (Fig. 2A). Type-I IFN levels were significantly higher for KS-infected animals than CA (*P* = 0.0152), BR, or PH (*P* < 0.0001) infected animals. KS-infected animals showed significantly higher levels of Type-II IFN than CA (*P* = 0.0401) as well as BR and PH (*P* = 0.0405 and *P* = 0.0431, respectively). Type-III IFN (IFNL) was higher in IAV than IBV-infected animals; however, only significant differences were found between BR and PH (*P* = 0.0074) (Fig. 2A). Additionally, TH1 (IP-10), TH2 (IL-2), and T-eff (IL-12p40) cytokines (Fig. 2B through D, respectively) were also significantly higher following IAV than IBV challenge (IP-10; CA to KS, BR, and PH [*P* < 0.0001] and KS to PH [*P* = 0.004]), (IL-2; CA and KS to BR and PH [*P* < 0.0001]), and (IL-12p40; CA and KS to BR and PH [*P* < 0.0001]). Pro-inflammatory cytokines and chemokines were also higher in IAV-challenged than in IBV-challenged animals. Pro-inflammatory response (Fig. 2E) was significantly higher for: (MCP-1; CA to KS [*P* = 0.0048] and BR and PH [*P* < 0.0001]), (MIP-1B; KS to BR [*P* = 0.0361]), and (TNFA; KS to BR [*P* = 0.0468]). Cytokine and chemokine levels over the entire 28-day period for each virus are shown in Fig. S2. Data from all analytes tested in sera show that for all groups (pro-inflammatory, TH1, TH2, T-effector, and IFN), IAV-challenged resulted in higher levels than IBV-challenged animals.

### HI and neutralizing antibody responses are delayed and reduced in ferrets challenged with IBV compared to IAV

**Fig 3:**
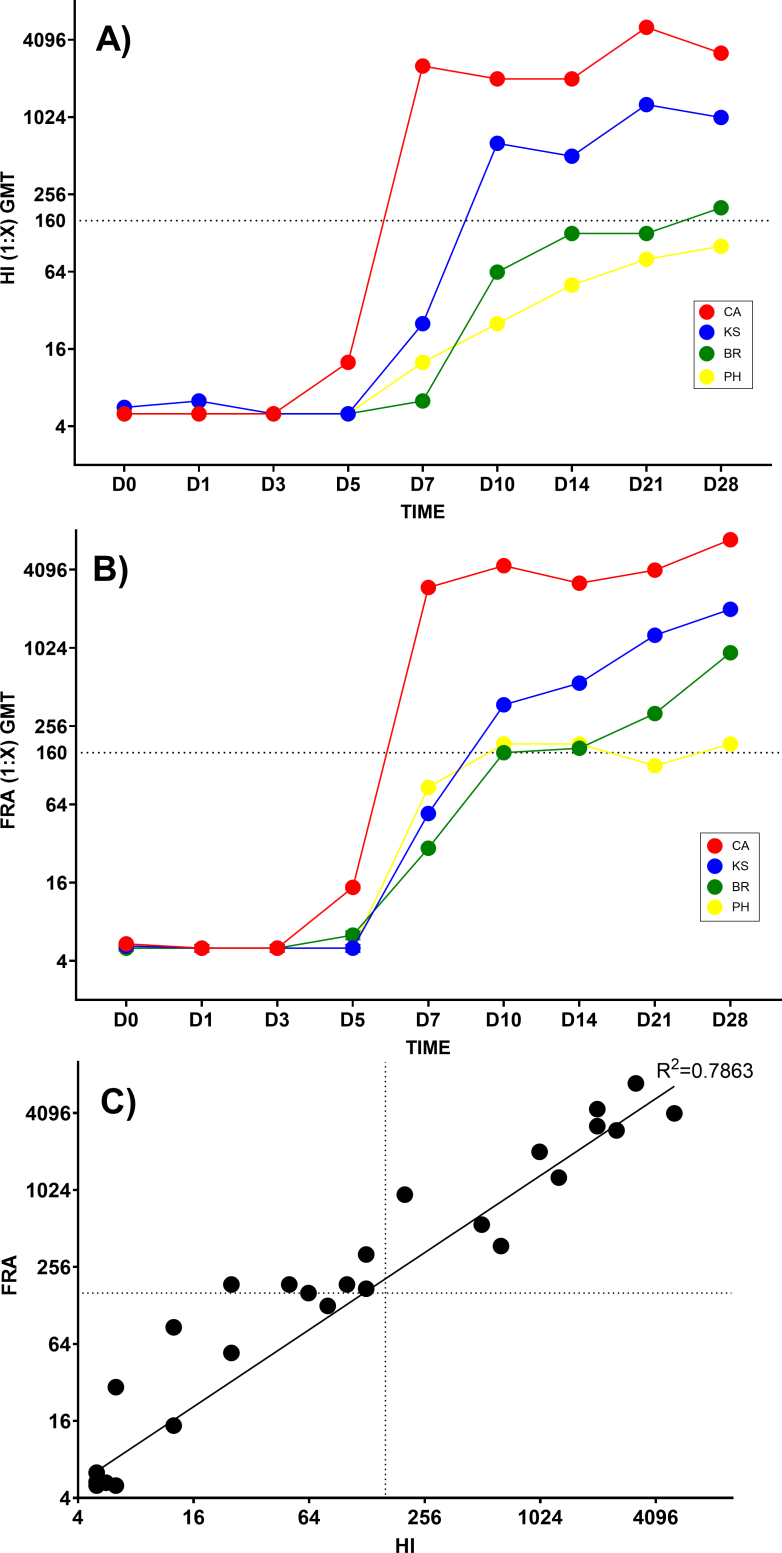
Serum antibody responses following IAV or IBV ferret challenge. Serum antibody by HI and neutralizing antibody (FRA) levels following infection with IAV (“CA” red, “KS” blue) and IBV (“BR” green, “PH” yellow) over 28 days post-challenge. All 18 ferrets tested at D0 and three ferrets/time point (D1–D28) were tested in triplicate for each assay. Average geometric mean titer (GMT) given for HI (**A**) and FRA (**B**) tests from values above 1:160 (dotted line) indicate robust antibody response. Correlation of HI to FRA (**C**).

Serum antibody responses were compared between IAV- and IBV-challenged animals for 28 days. The antibody responses detected by HI (Fig. 3A) showed clear differences between IAV (CA and KS) and IBV (BR and PH). For IAVs, infection with CA resulted in significantly greater antibody response than infection with KS (*P* = 0.0094; two-way ANOVA); however, for infection with IBVs no significant difference in antibody response was observed. Both IAVs showed significantly higher antibody responses to IBVs (CA vs BR/PH, *P* < 0.0001; KS vs BR, *P* = 0.0074 and KS vs PH, *P* = 0.0005). By HI, sera from IAV surpassed the 1:160 threshold by D7 (CA, HI = 2,560) and D10 (KS, HI = 640) post-challenge, whereas only one IBV (BR) surpassed the threshold but not until D28 post-challenge (HI = 201). Neutralizing antibody responses by FRA (Fig. 3B) mimicked antibody responses seen by HI; however, due to its greater sensitivity at lower antibody concentrations (Fig. 3C), both IBVs crossed the 1:160 threshold but not until very late post-challenge (BR on D21, FRA = 320; PH on D28, FRA = 186). Significant differences between both IAVs and either IBV, BR, or PH (*P* < 0.0001; two-way ANOVA) by FRA were observed. Antibody responses to infection with CA were also significantly higher than following KS infection (*P* < 0.0001). A strong correlation was observed (r^2^ = 0.786) between FRA and HI (Fig. 3C). Sera showed higher titers and greater sensitivity by FRA, especially at titers <1:160. Since a high correlation was observed, the FRA can be used as a confirmatory assay to the HI. Additionally, since the FRA measures neutralizing antibodies, this assay is ideal for detecting protective anti-influenza antibodies. Both IAV showed significantly higher neutralization titers compared to IBV for all viruses (*P* < 0.0001); however, no statistical differences between IBV (BR and PH) were observed. Results of antibody responses in ferrets showed that IAV initiated an earlier and higher neutralizing antibody titer that was sustained over the 28-day period. In contrast, IBV neutralizing antibody titers were delayed and failed to reach comparable titers to IAV by D28. Both HI and neutralizing antibody (FRA) responses in ferrets following IAV or IBV infection are consistent with antibody responses seen with previously tested viruses.

### Correlation between antibody responses and protein levels in ferret serum samples

We compared anti-influenza antibody titers in the serum to cytokine/chemokine levels (Tables 4 and 5) to determine whether there was a correlation between immune modulators to antibody levels following IAV and IBV infection. Serum samples (pre-challenge and Days 1, 3, 5, 7, 10, 14, 21 and 28 post-challenge), were tested to determine whether a correlation was seen when comparing antibody levels (HI and FRA) to analyte levels in ferret sera following IAV and IBV challenge (Tables 4 and 5). Direct correlations of serum HI (Table 4) and neutralizing FRA (Table 5) antibody was seen for all IAVs and IBVs to TH2 (IL-2) and T-effector (IL12-p40) analytes. These analytes indicated that strong adaptive (TH2 and T-effector) responses were associated with increased antibody responses. Interestingly, an inverse correlation between the pro-inflammatory response (MCP-1 chemokine) was observed for all IAVs and IBVs indicating that a strong pro-inflammatory response may dampen the antibody response. Some differences in correlation are seen comparing HI and FRA to analyte levels. For instance, IFNB showed a direct correlation to HI antibody response to all IAVs and IBVs (Table 4); however, by FRA, only IAV (KS) and IBV (PH) showed a direct correlation (Table 5). Also, T-effector (IL-4) showed an inverse correlation to all IAVs and IBVs by FRA (Table 5); however, this correlation was only observed for three of the four viruses by HI (Table 4). Correlations to IFNs are also seen depending on whether HI or FRA is used for the comparisons. For example, Type-III IFN (IFNL) levels showed direct correlation to IAV and inverse correlation to IBV using HI (Table 4) ; however, by using FRA, this is not a clear distinction (Table 5). Since analytes are secreted with different kinetics over the entire 28 days (Fig. S2), a clearer distinction could be obtained if the window of comparisons were narrowed. Overall differences in the analytes expression, as well as gene expression, showed differences in immune responses to IAVs and IBVs.

**TABLE 4:** Correlations between antibody levels (HI) and protein levels in ferret sera^*a*^

		CA (H1PDM09)			KS (H3N2)			BR (B-VIC)			PH (B-YAM)		
Group	Analyte	*r*	*P* value	Significance	*r*	*P* value	Significance	*r*	*P* value	Significance	*r*	*P* value	Significance
Interferon	IFNb	0.01703	0.4867	ns	0.6723	0.0272	*	0.08381	0.3889	ns	0.322	0.197	ns
	IFNg	−0.2178	0.2895	ns	−0.3846	0.1517	ns	0.1876	0.3128	ns	−0.2172	0.2857	ns
	IFNL	0.01703	0.4867	ns	0.06723	0.4342	ns	−0.3592	0.4444	ns	0.322	0.197	ns
TH1/TH2	IP-10	−0.315	0.2031	ns	−0.2605	0.2476	ns	−0.2885	0.2243	ns	0.09574	0.4026	ns
	IL-2	0.2949	0.2161	ns	0.6051	0.0454	*	0.5246	0.0734	ns	0.7659	0.0113	*
Chemokine	MCP-1	−0.5789	0.0544	ns	−0.7143	0.0181	*	−0.8569	0.003	**	−0.2666	0.2413	ns
	MIP-1b	−0.5363	0.0709	ns	−0.2101	0.2927	ns	0.04789	0.4537	ns	−0.2959	0.2183	ns
T-eff	IL-4	−0.2128	0.2907	ns	0.09244	0.4082	ns	0.3262	0.1907	ns	−0.6468	0.0339	*
	IL-17	−0.4341	0.121	ns	−0.2773	0.2326	ns	0.2011	0.3012	ns	−0.2785	0.2322	ns
	IL-12p40	0.6214	0.0411	*	0.4454	0.1151	ns	0.5596	0.0595	ns	0.6615	0.0303	*
	IL-12p70	−0.6895	0.0236	*	−0.5714	0.057	ns	0.4654	0.1021	ns	−0.3307	0.1903	ns
Pro-inflam.	TNFa	−0.5874	0.0516	ns	−0.3866	0.1517	ns	0.498	0.0884	ns	−0.2437	0.2621	ns
	IL-6	−0.03463	0.4673	ns	−0.3109	0.2061	ns	−0.853	0.0032	**	0.1567	0.343	ns
	IL-8	−0.2639	0.2451	ns	0.5042	0.0849	ns	−0.4984	0.086	ns	0.6267	0.0396	*

^*a*^ Spearman correlation of antibody response (HI) in serum to protein (analyte) levels in serum. Direct correlations (*r* > 0) and inverse correlations (*r* < 0) are indicated in “*r*” column. Significance (*P* < 0.05) correlations are indicated in Significance column by asterisks (*P < 0.05, **P < 0.01, ***P < 0.001); ns indiates no significance.

**TABLE 5:** Correlations between neutralizing antibody levels (FRA) and protein levels in ferret sera^*a*^

		CA (H1PDM09)			KS (H3N2)			BR (B-VIC)			PH (B-YAM)		
Group	Analyte	*r*	*P* value	Significance	*r*	*P* value	Significance	*r*	*P* value	Significance	*r*	*P* value	Significance
Interferon	IFNb	−0.1255	0.3753	ns	0.6781	0.0269	*	−0.4178	0.3333	ns	0.3281	0.1929	ns
	IFNg	−0.3889	0.1463	ns	−0.3103	0.2081	ns	0.3157	0.207	ns	0.5057	0.0833	ns
	IFNL	−0.1255	0.3753	ns	0.08476	0.4205	ns	−0.4178	0.3333	ns	0.3281	0.1929	ns
TH1/TH2	IP-10	−0.41	0.1363	ns	−0.2373	0.2711	ns	−0.2034	0.3031	ns	0.2039	0.2976	ns
	IL-2	0.4034	0.1392	ns	0.6103	0.0454	*	0.3899	0.1512	ns	0.8335	0.0052	**
Chemokine	MCP-1	−0.6444	0.0343	*	−0.8815	0.0017	**	−0.8476	0.0035	**	−0.3784	0.154	ns
	MIP-1b	−0.795	0.0072	**	−0.2034	0.3031	ns	0.13	0.3733	ns	−0.1064	0.3929	ns
T-eff	IL-4	−0.3682	0.1632	ns	−0.1187	0.3855	ns	−0.06781	0.4372	ns	−0.4256	0.1258	ns
	IL-17	−0.7615	0.011	*	−0.2034	0.3031	ns	0.2543	0.2573	ns	−0.1419	0.3579	ns
	IL-12p40	0.569	0.0576	ns	0.4577	0.1106	ns	0.4916	0.0925	ns	0.7625	0.0123	*
	IL-12p70	−0.9121	0.0007	***	−0.4238	0.1295	ns	0.3745	0.1586	ns	−0.1064	0.3929	ns
Pro-inflam.	TNFa	−0.6695	0.0275	*	−0.3221	0.2	ns	0.431	0.1233	ns	−0.08867	0.4119	ns
	IL-6	0.1702	0.3305	ns	−0.2373	0.2711	ns	−0.8728	0.001	***	0.2926	0.2187	ns
	IL-8	−0.5941	0.0493	*	0.5933	0.0511	ns	−0.339	0.1867	ns	0.3901	0.1456	ns

^*a*^ Spearman correlation of neutralizing antibody response (FRA) in serum to protein levels in serum. Direct correlations (*r* > 0) and inverse correlations (*r* < 0) are indicated in “*r*” column. Bold. Significant (*P* < 0.05) correlations are indicated in Significance column by asterisks (*P < 0.05, **P < 0.01, ***P < 0.001); ns indicates no significance.

## DISCUSSION

This study was designed to determine whether innate immune markers and soluble cytokines/chemokines in serum following challenge could shed light on why IBVs do not elicit an antibody response as quickly and robustly as IAVs in ferrets. Four representative viruses were used in this study, two IAVs and two IBVs, to determine innate and adaptive differences following challenge in ferrets. Initial differences in clinical signs as well as virus kinetics following IAV or IBV were apparent. Clinically, IAVs caused greater lethargy (average RII = 1.31) compared to IBV (average RII = 1.03), greater maximum weight loss (IAV ~6% and IBV ~4%), and more sneezing (IAV ~52%; IBV ~33%) during viremia. In addition to greater morbidity, IAVs virus replication peaked early (D1 post-challenge) compared to IBV (D3 post-challenge) in ferrets. These observations could indicate that IAVs initiate an earlier innate immune response than IBVs which could lead to an earlier and more robust adaptive immune response. It has been shown for COVID-19 vaccination that a strong innate immune response results in an early, robust antibody response (42).

In ferret primary respiratory tract cells, pro-inflammatory genes and IFNs are delayed and downregulated by IBVs compared to IAVs (31). TH1 (*CXCL10*), TH2 (*IL-2*), and T-effector (*IL-4*) were also significantly upregulated by IAV. Similar responses were seen in the URT and PBMC from IAV- and IBV-infected ferrets in this study. IAV (CA), in particular showed early (D3) upregulation of TH1 (CXCL10), and late (D5) upregulation of TH2 (*IL-2*) and T-effector (*IL-4*) in the URT indicating a switch to a strong adaptive immune response. For IFN response in the URT, only Type-II (*IFNG*) and Type-III (*IFNL3*) IFNs were upregulated from pre-challenge levels following IAV (CA) challenge. In PBMC by D5 post-challenge only T-effector (*IL-4*) and Type-III IFN (*IFNL3*) were upregulated by IAV (KS). However, early D3 upregulation TH1 (*CXCL10*) was highest for IBV (PH) which switched to late D5 highest upregulation for IAV (KS). Interestingly IFN response (*SOCS3*) gene, which is responsible for downregulating the IFN response was highest for IBV (BR) both early and late post-challenge. This may support that IBV has greater IFN-antagonism properties than IAV. Unfortunately, due to the limited number of samples tested (three animals per time point), significant differences for gene expression in all genes were not possible. Additional studies with greater samples collected and tested at each time point may aid in increasing the significance as was observed using ferret respiratory epithelial cells (31). However, it is important to note that the responses seen in this study showed similar trends as observations from respiratory epithelial cells infected with IAV and IBV.

In both HI and neutralizing antibody tests, IAV infections generated an early, strong antibody response between Days 7 and 10 post-challenge, whereas IBV infections did not reach equivalent HI titers and required at least 21–28 days to reach titers of at least 1:160 by FRA. There is a clear delay compared to antibody responses following IAV challenge. Antibodies to IAV may persist for up 18 months or longer in humans (43–45); however, the longevity of IBV directed antibodies has not been tested. Differences in immunogenicity of HA proteins of IBV compared to IAV may have an effect on antibody responses, however, this could not be ascertained in this study.

To determine whether there were differences in cytokine and chemokines levels in ferrets following IAV and IBV infection over 28 days, we tested sera by multi-plex or bioassay (IFNL). IAV infection resulted in significantly higher levels of Type-I/II IFNs, TH1 (IP-10), TH2 (IL-2), T-effector (IL-12p40), and pro-inflammatory cytokines/chemokines (MCP-1, MIP-1B, and TNFA) that were correlated to increases in antibody responses. This further supports the assumption that a strong innate immune response will lead to a robust antibody response.

Further exploration into why IBVs generate weaker antibody responses compared to IAVs in ferrets could be linked to the proposed mammalian reservoir for these viruses. Since IBV has a host range limited to mammals, it may be more effective at evading the host’s primary immune responses and the absence of immune pressures exerted by non-mammalian hosts (46). Overall, IBVs have a greater suppression of innate immunity than IAV which may lead to a reduction in antibody responses. Suppression of the IFN response is a hallmark of immune evasion by influenza viruses (47). Since IBV NS1 has been shown to interfere with human IFN signaling by preventing activation of upstream transcription factor IRF-3 (48) and targeting host ISG15 (49), further exploration of IBV on host IFN responses is warranted. Use of effective IFN adjuvants to improve innate responses may increase antibody responses to IBV as well as reduce morbidity. This information may be important in developing improved vaccines for influenza viruses.

## SUPPLEMENTAL MATERIAL

Fig. S1 mbio.02361-24-s0001.pdf Temperature and weight changes in ferrets following influenza challenge.

Fig. S2 mbio.02361-24-s0002.pdf Kinetics of cytokines and chemokines in serum.

Table S1 mbio.02361-24-s0003.pdf Ferret primers and probes.

ASM does not own the copyrights to Supplemental Material that may be linked to, or accessed through, an article. The authors have granted ASM a non-exclusive, world-wide license to publish the Supplemental Material files. Please contact the corresponding author directly for reuse.
