# Supplementary material for: Delay of innate immune responses following influenza B virus infection affects the development of a robust antibody response in ferrets
Source: mBio. 2025 Jan 8;16(2):e02361-24. doi: 10.1128/mbio.02361-24 (PMC11796412; doi:10.1128/mbio.02361-24)
Supplement: Fig. S1 — Temperature and weight changes in ferrets following influenza challenge. [file mbio.02361-24-s0001.pdf]

Supplemental Figure S1: Temperature and weight changes in ferrets following influenza challenge.

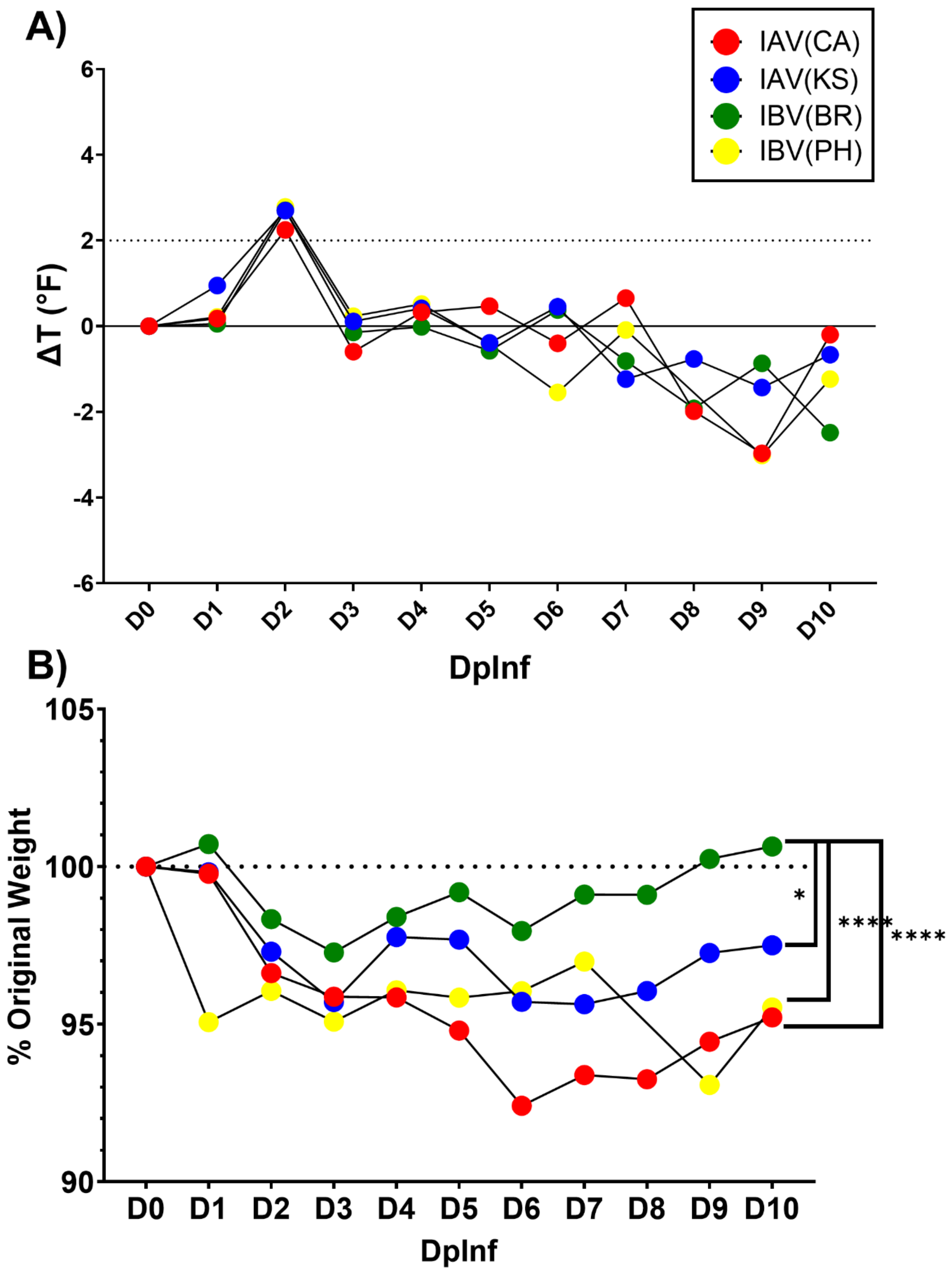

Clinical parameters in ferrets challenged with IAV and IBV. Red (IAV, H1N1pdm09subtype, CA), blue (IAV, H3N2 subtype, KS), green (IBV, Victoria lineage, BR), yellow (IBV, Yamagata lineage, PH). A) Average body temperature changes from baseline following challenge. Fever indicated by dotted line at +2°F above baseline. B) Weight loss following challenge. Percentage of original, pre-challenge, weight. Significant differences between IAV (CA/KS) and BR as well as PH and BR (2-way ANOVA).
