# Supplementary material for: Delay of innate immune responses following influenza B virus infection affects the development of a robust antibody response in ferrets
Source: mBio. 2025 Jan 8;16(2):e02361-24. doi: 10.1128/mbio.02361-24 (PMC11796412; doi:10.1128/mbio.02361-24)
Supplement: Fig. S2 — Kinetics of cytokines and chemokines in serum. [file mbio.02361-24-s0002.pdf]

Supplemental Figure S2: Kinetics of Cytokine and Chemokines in Serum.

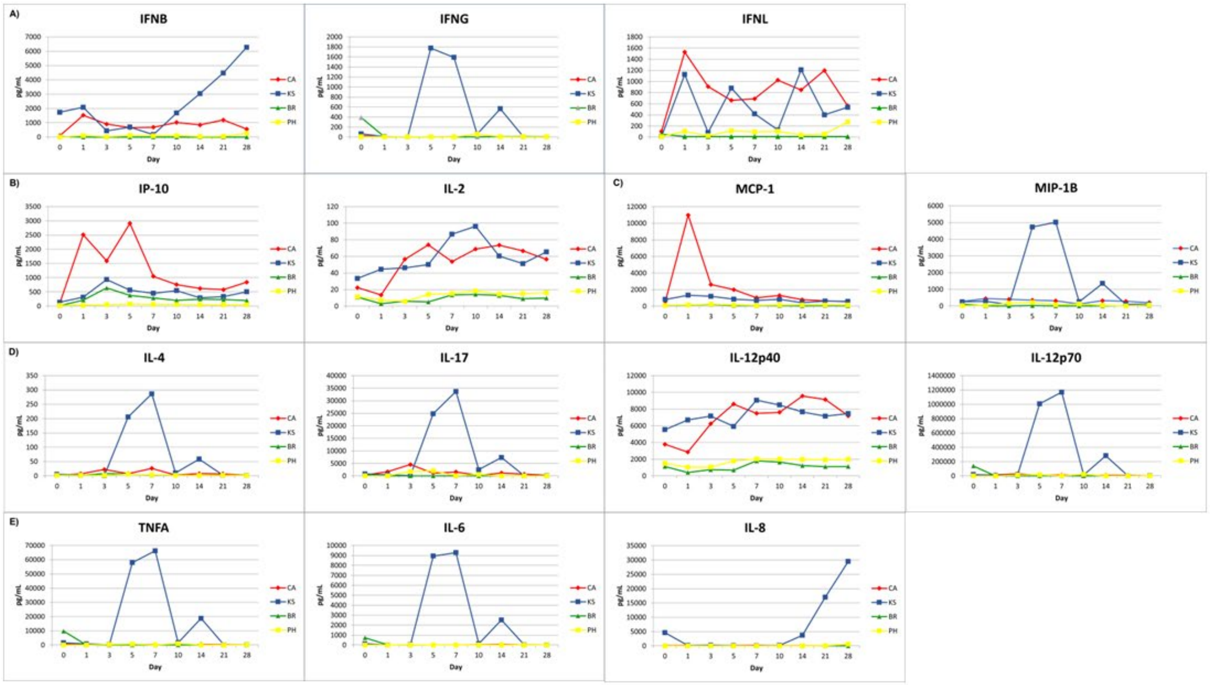

A) Interferon responses. Type-I (IFNB), Type-II (IFNG) and Type-III* (IFNL). *Bio-assay B) TH1 (IP-10) and TH2 (IL-2) response C) Pro-inflammatory chemokines (MCP-1 and MIP1B) D) T-effector response (IL-4, IL-17, IL-12p40 & IL-12p70) E) Pro-inflammatory cytokine response (TNFA, IL-6, and IL-8). N=3 ferrets/timepoint.
