# Supplementary material for: Delay of innate immune responses following influenza B virus infection affects the development of a robust antibody response in ferrets
Source: mBio. 2025 Jan 8;16(2):e02361-24. doi: 10.1128/mbio.02361-24 (PMC11796412; doi:10.1128/mbio.02361-24)
Supplement: Table S1 — Ferret primers and probes. [file mbio.02361-24-s0003.pdf]

Supplemental Table S1: Ferret primers and probes.

GENE TARGET	FORWARD PRIMER 5'→3'	REVERSE PRIMER 5'→3'	PROBE: (5' CHEMISTRY), <MODIFIED BASE>, (3' CHEMISTRY)	USE	REFERENCE	FUNCTION
GAPDH	TGCGGCCAAGGCAGTAG	AGGCCATGCCAGTGAGCTT		SYBR green	Carolan et al.	Housekeeping gene
MCP1	GCAGCAAGTGTCCCAAAGAAG	GACTGGGGTCAGCGCAGAT	FAM-ATCCTCAAGAC<LNA A>TTCCT-BHQ1	TaqMan	Carolan et al.	Inflammatory
CXCL10	CCTGGCTTCACCGAGTTCT	AGTAGCAGCCCATGGAGTAAAA		SYBR green	Maines et al.	Th1
IL-2	GTTAAAAATTATGAGAGCCCCAGGA	TTGAGTTCTTCTGCTAGACATTGAAGA	FAM-CTAC<LNA A>TGCCCAAGAAG-BHQ1	TaqMan	Carolan et al.	Th2
TGFB1	CGTGCGGCAGCTCTATATT	GCAGAAATTGGCGTGGTAAC	FAM-AAGGATCTGGGCTGGAAGTGG<LNA A>ATC-BHQ1	TaqMan	Rowe et al.(2024)	Treg
IL-4	CCAACAGATTGCTCAGAGGACTT	CACCGAACAGGTCATGTTTGC	FAM-CAGGAACCTC<LNA A>GGAACAT-BHQ1	TaqMan	Carolan et al.	Teff
IL-12p40	GGTGCTATTCACAAGCTCAAGTATG	GGTTTGATGATGTCCCTGATGA	FAM- TACACCAGC<LNA A>GCTTC-BHQ1	TaqMan	Carolan et al.	Teff
IL-17	GGACGGTAAACTACCACATGAACTC	AGACTCCCTTCGCAGAACCA	FAM-TCCCC<LNA A>TCCAGCAAGA-BHQ1	TaqMan	Carolan et al.	Teff
IL-1B	CCTGGTGCTGTATAACTCGTATGAG	TTGGTTCACACTAGTTCCGTTGA	FAM-TCGGGCGCTCC<LNA A>C- BHQ1	TaqMan	Carolan et al.	Pro-inflammatory
IL-6	GCAGAGAACAACCTAAATCTTCCAA	TGATTGAATTGAGACTGGAAGCA	FAM-CTGGC<LNA A>GAAGAGGAC-BHQ1	TaqMan	Carolan et al.	Pro-inflammatory
Granzyme A	GGATCCTCCCTCTCCCTAAGAA	CCCAGCCTGCAACTTGACA	FAM-ATG<LNA A>TGTCAAACCCGAAAC-BHQ1	TaqMan	Carolan et al.	Apoptosis
IFNA	TCCATCCTGAGGAACTACTTCCAG	AGGCACAAGGGCTGTATTGC	FAM-GAATCTCCCTCT<LNA A>TCTGC-BHQ1	TaqMan	Carolan et al.	IFN (Type I)
IFNB	ATATTTCTCCACCACGGTTCTTG	ACTCCACACTGCTGCTGCTTAG	FAM-AACTATAACTT<LNA A>CTTCGATTCCA-BHQ1	TaqMan	Carolan et al.	IFN (Type I)
IFNG	AACTGGAGAGAGGAGAGTGACAAAA	GTCTTCCTTGATGGTATCCATGC	FAM-TCTCCTTCT<LNA A>CTTGAAACTGT-BHQ1	TaqMan	Carolan et al.	IFN (Type II)
IFNL3	CCAGCCCTGCCTTAAGTTATT	CCTTCCTGTTTACTTGTGCATATTG	FAM-ATGAAACCAG<LNA A>GTGCTGACCCAAA-BHQ1	TaqMan	Rowe et al. (2024)	IFN (Type III)
STAT1	AGCCTTGCATGCCAACTCA	ACAGTCCAGCTTCACCGTGAA		SYBR green	Fang et al.	IFN response
STAT2	AGCTGCTGAAGGAGCTGAAG	TGCCTTCCTGGAGTCTCACT		SYBR green	Fang et al.	IFN response
STAT3	CAACCCCAAGAACGTGAACT	AGCCCACGTAATCTGACACC		SYBR green	Fang et al.	IFN response
RIG-I	AGAGCACTTGTGGACGCTTT	TGCAATGTCAATGCCTTCAT		SYBR green	Fang et al.	IFN response
SOCS3	GCTGGTGCATCACTACATGC	GACCGTCTTCCGACAGAGAT		SYBR green	Fang et al.	IFN response
TSLP	GTCTGGGCACATAACTCTAAGG	CACCCTGGTGTCTCACTAAAC	<FAM>CAGGCCTTGC<LNA A>GATATAGAGCCGATT<BHQ1>	TaqMan	Rowe et al.(2024)	IFN response

List of qRT-PCR primers. All primers used in this study are referenced. Forward (5' to 3'), reverse (5' to 3') and probes are listed. Probes using TaqMan enzyme include special chemistry at the 5'-end (FAM) 3'-end (BHQ1) and internally modified bases (LNA A) that were specifically designed for this study to enhance binding and specificity for ferret genes.
